# The Incident of Multiple Skin Necrosis and Unilateral Vision Loss Post Liposuction: A Case Report

**DOI:** 10.7759/cureus.40384

**Published:** 2023-06-13

**Authors:** Khalid A Fayi, Hassan A Ali, Nashwa M Ali

**Affiliations:** 1 Plastic and Reconstructive Surgery Section, Department of Surgery, King Faisal Specialist Hospital and Research Centre, Riyadh, SAU; 2 Department of Plastic and Reconstructive Surgery, King Abdulaziz Medical City, Riyadh, SAU; 3 College of Medicine, Alfaisal University, Riyadh, SAU

**Keywords:** thrombosis, vision loss, skin graft, debridement, liposuction, skin necrosis, multiple skin necrosis

## Abstract

For many years, people with excess weight around specific body parts who wanted to improve their shape or establish symmetry to achieve their ideal body image chose liposuction. As with any intervention, there is a chance of complications and unfavorable outcomes with liposuction. As a late result of the procedure, skin necrosis, infection, and hematoma are some of the known complications of such a procedure. Other known complications include damage to surrounding structures like nerves, vasculature, or perforating body viscera. This study aimed to report an eventful and unwanted result of a common and relatively safe cosmetic procedure. A 31-year-old Saudi female presented to the emergency room (ER) with right unilateral vision loss, bruises, and burning pain involving the upper limbs, thighs, abdomen, back, and flanks after having liposuction and rhinoplasty two weeks ago in a private clinic overseas. Multiple investigations were obtained to investigate her blindness, which showed a right upper branch of retinal vein occlusion. She was treated conservatively with daily wound dressings and analgesics. After five days, the patient returned with infected wounds and clinical deterioration, necessitating multiple excisions, debridement, and grafting. She eventually recovered and was discharged in good health. Herein, we report a rare case of unilateral blindness and multiple skin necrosis following liposuction of the abdomen and thigh. Debridement and skin grafting were the ideal treatment strategies.

## Introduction

One of the most popular cosmetic procedures today is liposuction [1]. Liposuction is a surgical procedure that removes subcutaneous fat by inserting an aspiration cannula through minute skin incisions and using suction to help [2].

The hazards involved with liposuction can be readily neglected because it is a minimally invasive, relatively safe procedure that can be carried out in outpatient clinics [3]. Contour deformity, with a reported frequency of 20%, is the most prevalent complication, followed by seroma, hyperpigmentation, asymmetry, and a hypertrophic scar [4]. The overall complication rate has been reported to be in the range of 8.6-20% [5].

In 0.02-0.25% of cases, major or fatal consequences like skin necrosis, infection, necrotizing fasciitis, pulmonary embolism, and even death have been documented [6]. We present here a rare case report of unilateral blindness and multiple skin necrosis following liposuction of the abdomen and thigh, which was treated with debridement and a skin graft.

## Case presentation

**Figure 1:**
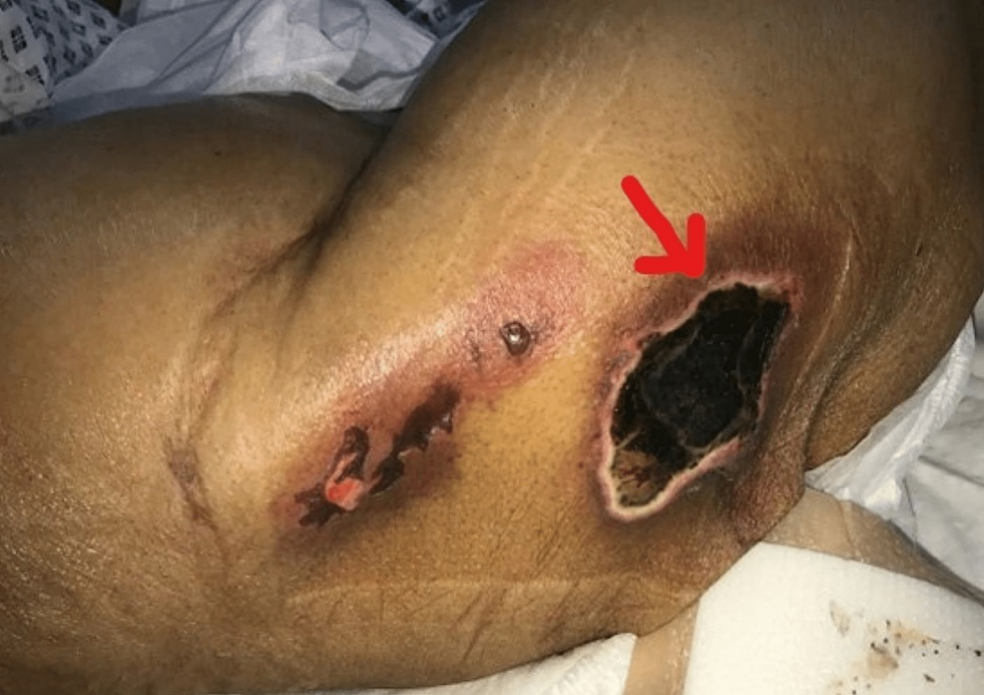
Bruises at areas where the liposuction was carried out.

A 31-year-old Saudi female patient presented to the emergency room (ER) with the following symptoms and signs: unilateral vision loss in the right eye, bruises, and burning pain involving the upper limbs, thighs, abdomen, back, and flanks. After stabilizing the patient by following the Airway, Breathing, and Circulation (ABC) protocol, a history and physical examination were obtained. History revealed that the patient is a smoker, on oral contraceptive pills, had a previous history of abortion, and underwent liposuction and rhinoplasty two weeks ago in a private clinic overseas as an affordable option for her. The affected areas were where the liposuction was carried out (Figure 1). The funduscopy showed bilateral severe optic disc swelling, more on the right side, which later improved. Additionally, a few Roth spots were observed in the right eye with a normal macula.

The patient was admitted to the burn ICU under the plastic surgery service for further assessment of her wounds and management. Multiple investigations, including hemoglobin (Hb: 107), white blood cell (WBC: 11.2) count, red blood cell (RBC: 4.37) count, and coagulation profile (partial thromboplastin time (PTT) 25, prothrombin time (PT) 10, international normalized ratio (INR) 1), were obtained. Hypercoagulabilities, such as protein C/S deficiency and antiphospholipid, were excluded. Cultures were done to rule out infectious processes and showed few non-specific Gram-positive bacteria. Imaging modalities, such as computerized tomography (CT) and magnetic resonance imaging (MRI), were ordered to investigate her blindness, which showed thrombosis of the right upper branch of the right retinal vein.

During her admission, the vision in her right eye started to improve following conservative management, acetazolamide, enoxaparin, and vitamins and minerals replacement. Following investigations, her primary physician decided to treat her conservatively with daily wound dressings and analgesics for the pain.

**Figure 2:**
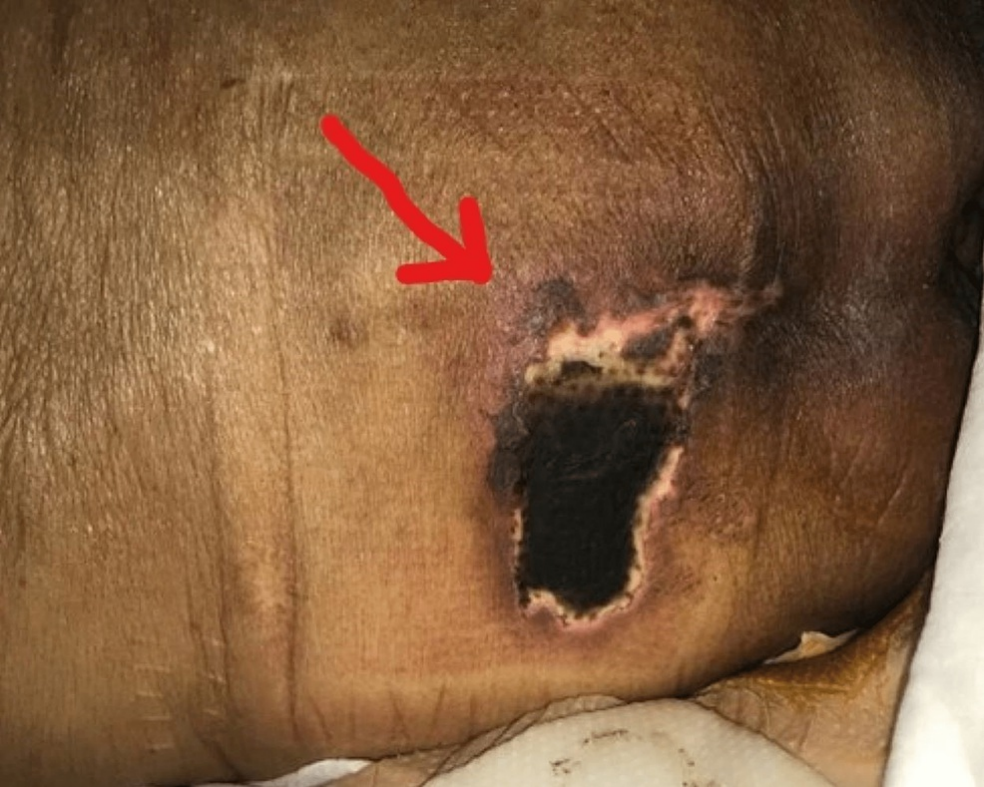
The wound after 12 days following daily wound dressings.

**Figure 3:**
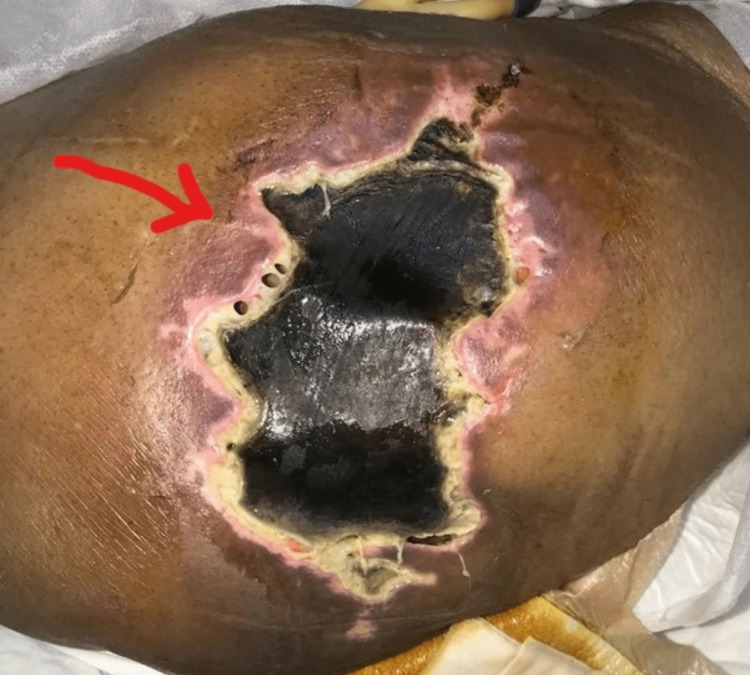
The wound upon second admission.

She was discharged in a stable condition after a 12-day hospital stay and a clinic follow-up with multiple specialties (Figure 2). After five days, the patient returned with a deteriorating condition and infected necrotic surgical sites where liposuction was performed and required surgical intervention (Figure 3).

She was readmitted, and the decision was made to perform multiple excisional debridements and grafting of the affected areas. 

The patient stated that she was discharged one day following the procedure with no scheduled follow-ups with the surgeon. She traveled back home and started noticing progressive bruising over the liposuction sites. After undergoing multiple debridements and skin grafting and being evaluated by a multidisciplinary team, she improved and was discharged home in a stable condition.

## Discussion

Since Charles Dujarier attempted to remove the excess fat from a dancer's legs in 1921, there has been a nearly century-long search to reduce the body's accumulation of excess fat [7]. The patient ended up needing to have his leg amputated because the procedure had injured his femoral artery, which also marked the beginning of the difficulties. In 1972, German doctor Schrudde began employing a uterine curette, a less intrusive method of fat removal. With his extensive series of cases, Grazer [8] later popularized liposuction (suction-assisted lipectomy).

Local and systemic liposuction complications can be distinguished. Contour abnormalities, seroma, hematoma, hypertrophic scar, and asymmetry are a few examples of the local consequences. The dangers to one's life are those associated with systemic issues. Bowel perforation, deep vein thrombosis, skin necrosis, sepsis, fat embolism, and hypothermia are among the common systemic consequences [9]. Herein, we present a 31-year-old Saudi female who presented to the ER with unilateral vision loss in the right eye, bruises, and burning pain involving the upper limbs, thighs, abdomen, back, and flanks after liposuction and rhinoplasty.

The rapid loss of vision known as ischemic optic neuropathy (ION) is brought on by a disruption in the blood supply to the optic nerve [10]. A few examples of ION following abdominal liposuction have recently been documented [11-17]. Minagar et al. [11] were the first to report ION following liposuction. Since then, a few patients with comparable clinical histories have been documented. After performing liposuction surgery, Foroozan and Varon [12] described a case of bilateral AION and hypothesized that the cause was due to the removal of a substantial volume of fat and the subsequent hemodynamic instability. Although ION occurs infrequently, hemodynamic instability and hypotension can develop in a variety of surgical and nonsurgical circumstances. Possible theories include a constellation of various causes or a single predisposing factor [13]. Another instance described by Ribeiro Monteiro et al. [14] proposed that a higher CSF pressure-related sensitivity to optic disc perfusion was the predisposing factor. Because the demographics of patients with idiopathic intracranial hypertension (IIH) and those who chose aesthetic abdominal surgery are similar, they recommended general screening for the presence of optic disc edema before undergoing liposuction procedures. According to Moura et al. [15], the predisposing factor for ION in their patient was a tiny, packed optic disc in the context of perioperative blood loss and overhydration. According to the case of a patient who had post-liposuction bilateral posterior ION, the methylenetetrahydrofolate reductase (MTHFR) homozygous and heterozygous to prothrombin II variant mutation was postulated to be the underlying cause of the optic nerve infarction [16]. Abri Aghdam et al. [17] recently described a 30-year-old girl with optic disc drusen (ODD) who underwent surgery, including abdominoplasty and liposuction, and afterwards acquired anterior ION (AION). The constellation of clinical results presented in this research is new. Regarding our case, the investigations obtained showed thrombosis. During her admission, her right eye improved. The use of MRI should not needlessly delay the initiation of aggressive surgical treatment, and CT scanning can be a more effective and rapid imaging test.

Sharp cannula tips that point upwards, as well as extensive superficial liposuction, increase the risk of skin necrosis by injuring the vascular subdermal plexus [9].

With 500 mg of lidocaine and 1 mg of epinephrine per liter, the recommended amount of infiltration for liposuction ranges from 1 to 3 L per liter of adipose tissue extracted [18]. In our case, probably a large amount of adrenaline was infiltrated during liposuction to shorten the time of the procedure, reduce bleeding during and after the procedure, and speed up the recovery, which may be the cause of skin necrosis.

Once necrosis is identified, it is treated with surgery, antibiotics, and hyperbaric oxygen therapy [19]. Early on, when skin erythema is present, both intradermal oxygen injection and hyperbaric oxygen therapy have been reported to be beneficial [20]. Regarding our case, firstly, she was treated with daily wound dressings and analgesics, but she returned with signs of infected wounds and clinical deterioration, and she was diagnosed with skin necrosis. Once skin necrosis was confirmed, the decision was made to perform excisional debridement and grafting of the affected areas in multiple procedures. The patient improved and was discharged in a stable condition after undergoing multiple procedures for debridement and skin grafting with the participation of other multidisciplinary teams.

## Conclusions

Skin necrosis is a rare surgical complication of liposuction; however, it poses a significant challenge for both the patient and the physician. Herein, we present a rare incident of multiple skin necrosis following liposuction of the abdomen and thigh. Debridement and skin grafting were the ideal treatment strategies. Additionally, our case was admitted with a rare occurrence of unilateral vision loss in the right eye, which improved during her hospitalization.
